# Inhibitory effect of *Selaginella doederleinii* hieron on human cytochrome P450

**DOI:** 10.3389/fphar.2023.1108867

**Published:** 2023-02-15

**Authors:** Fei Lin, Xinhua Lin, Xuewen Wang, Guanghui Mei, Bing Chen, Hong Yao, Lingyi Huang

**Affiliations:** ^1^ Department of Pharmaceutical Analysis, School of Pharmacy, Fujian Medical University, Fuzhou, China; ^2^ Department of Preventive Medicine, School of Public Health, Fujian Medical University, Fuzhou, China

**Keywords:** herb-drug interaction, the ethyl acetate extract from *S. doederleinii*, CYP inhibition, cocktail CYP450 assay, time--dependent inhibition, delicaflavone, amentoflavone

## Abstract

**Introduction:** *Selaginella doederleinii* Hieron is a traditional Chinese herbal medicine, the ethyl acetate extract from *Selaginella doederleinii* (SDEA) showed favorable anticancer potentials. However, the effect of SDEA on human cytochrome P450 enzymes (CYP450) remains unclear. To predict the herb-drug interaction (HDI) and lay the groundwork for further clinical trials, the inhibitory effect of SDEA and its four constituents (Amentoflavone, Palmatine, Apigenin, Delicaflavone) on seven CYP450 isoforms were investigated by using the established CYP450 cocktail assay based on LC-MS/MS.

**Methods:** Appropriate substrates for seven tested CYP450 isoforms were selected to establish a reliable cocktail CYP450 assay based on LC-MS/MS. The contents of four constituents (Amentoflavone, Palmatine, Apigenin, Delicaflavone) in SDEA were determined as well. Then, the validated CYP450 cocktail assay was applied to test the inhibitory potential of SDEA and four constituents on CYP450 isoforms.

**Results:** SDEA showed strong inhibitory effect on CYP2C9 and CYP2C8 (IC_50_ ≈ 1 μg/ml), moderate inhibitory effect against CYP2C19, CYP2E1 and CYP3A (IC_50_ < 10 μg/ml). Among the four constituents, Amentoflavone had the highest content in the extract (13.65%) and strongest inhibitory effect (IC_50_ < 5 μM), especially for CYP2C9, CYP2C8 and CYP3A. Amentoflavone also showed time-dependent inhibition on CYP2C19 and CYP2D6. Apigenin and Palmatine both showed concentration-dependent inhibition. Apigenin inhibited CYP1A2, CYP2C8, CYP2C9, CYP2E1 and CYP3A. Palmatine inhibited CYP3A and had a weak inhibitory effect on CYP2E1. As for Delicaflavone, which has the potential to develop as an anti-cancer agent, showed no obvious inhibitory effect on CYP450 enzymes.

**Conclusion:** Amentoflavone may be one of the main reasons for the inhibition of SDEA on CYP450 enzymes, the potential HDI should be considered when SDEA or Amentoflavone were used with other clinical drugs. On the contrast, Delicaflavone is more suitable to develop as a drug for clinical use, considering the low level of CYP450 metabolic inhibition.

## 1 Introduction

Numerous studies have shown that drug-drug interaction (DDI) can cause serious adverse reactions, drug ineffectiveness and even death (Han et al., 2012; Kozakai et al., 2012; Al-Ramahi et al., 2015; Guengerich, 2021). Induction or inhibition of human Cytochromes P450 (CYP450) enzyme by compounds is the main cause for drug-drug metabolic interaction (Qin et al., 2013). About 60%–70% of the clinical drugs are clear by phase I reaction, where 75% of the involved metabolizing enzymes are CYP450 isoforms (Kim et al., 2005; Lee and Kim, 2013). Usually, CYP450 inhibition leads to drug accumulation and even toxicity (Lin and Lu, 1998; Guengerich, 2008; Hakkola et al., 2020). Based on that, DDI test is necessary for a chemical from pre-clinical stage into clinical trials in pharmaceutical industry. The United States Food and Drug Administration (FDA) guidelines for DDI test have recommended several major human CYP450 isoforms, including CYP1A2, CYP2C8, CYP2C9, CYP2C19, CYP2D6 and CYP3A. Those CYP450 isoforms have account for mostly enzymatic metabolism of clinical drugs (Prueksaritanont et al., 2013; Sudsakorn et al., 2020). Although CYP2E1 was not clearly specified in the FDA guidelines, many studies have revealed that CYP2E1 can metabolize Coumarin, Quinoline, Isoniazid, ethanol, Caffeine, acetaminophen and other substances. Therefore, it is reasonable to include CYP2E1 when conducting the DDI study (Valicherla et al., 2019).

Nowadays, many people consume herbal products for self-health or medical use in their daily life. Similar to small molecule medicine, when people take herbal products and drugs at the same time, potential herb-drug interaction (HDI) should be considered as well (van Breemen, 2015). In the past 20 years, more and more studies have shown that there are significant CYP450 enzyme interaction between herbal products and prescription medicines, which will increase patients’ risk of unnecessary and unintended adverse effect. For example, *Angelica dahurica* root extract inhibited CYP450 enzymes such as CYP2C, CYP3A and CYP2D1, and caused pharmacokinetic and pharmacodynamic interactions with Tolbutamide and Diazepam (Ishihara et al., 2000). Shenmai injection, one of the most popular herbal medicines in China, has reported to inhibit CYP3A1/2 and CYP2C6 (Xia et al., 2010). Researchers have found that Huanglian [*Rhizoma coptidis* (L.)] has obvious inhibition on CYP2D6 (Han et al., 2011). *Newbouldia laevis* extract inhibited the enzyme activities of CYP1A2, CYP2C9, CYP2C19, and *Newbouldia laevis* showed time-dependent inhibition effect on CYP1A2 (Thomford et al., 2016). Licorice is a common use dietary supplement even is consumed as a condiment, which was found to inhibit several CYP450 enzymes. What’s more, three major licorice species (*G. glabra, G. uralensis* and *G. inflata*) showed unique pattern pf enzyme inhibition (Li et al., 2017). The most reported CYP450 isoforms involved in HDI are CYP1A2, CYP2C, CYP2D6 and CYP3A, which is similar to the list of small molecules DDI test according to FDA guideline (Choi et al., 2016; Awortwe et al., 2018).

*Selaginella doederleinii* Hieron belongs to *Selaginella* genus of the Selaginellaceae family and is commonly used as a traditional Chinese herbal medicine, which was reported to have anti-hyperglycemia, anti-virus, anti-cancer and other pharmacological activities, and used in the treatment of nasopharyngeal carcinoma, lung cancer and trophoblastic tumor (Zheng et al., 2005; Chen et al., 2018). The previous *in vitro* and *in vivo* studies have revealed that the ethyl acetate extract from *S. doederleinii* (SDEA) had anti-cancer effect (Yao et al., 2017), especially for lung and colorectal cancer (Sui et al., 2016; Li et al., 2020). Those main ingredients from the extract, such as Amentoflavone, Palmatine, Apigenin and Delicaflavone, may account for the pharmacological activities of SDEA. Among those components, Delicaflavone was proved to induce apoptosis in cervical cancer HeLa cells (Yao et al., 2019), colorectal cancer cells (Yao et al., 2020) and lung cancer cells (Sui et al., 2017). Moreover, Delicaflavone can reverse cisplatin resistance via endoplasmic reticulum stress signaling pathway in non-small cell lung cancer cells, which may serve as a useful adjunct in treatment of cisplatin-resistant lung cancer (Wang et al., 2020). Therefore, SDEA and Delicaflavone are the most promising anti-tumor agents, whether use alone or in combination with other drugs.

Although the pharmacological activities of SDEA and its bioactive compounds have been extensively studied, there is no report on HDI of SDEA on human CYP450 enzymes. As a plant extract, the composition of SDEA is complex, whose metabolic process in human body is not fully understood. Meanwhile, the HDI study for the bioactive components of SDEA have not been conducted either. The pre-clinical study on the ADME properties of candidate compound is of great significance for improving success rate, reducing cost and toxic risk, which is crucial for further drug development. This rule also applies to herbal product drug development (Brantley et al., 2014). Regulatory agencies in most countries, such as FDA, EMA, NMPA, et al., all require that new chemicals should be clarified the possible inhibition or induction effect on human CYP450 enzyme in the investigational new drug (IND) application stage. For newly developed medicines, they are likely to be accepted on the market only if they have clear human metabolic information (Sudsakorn et al., 2020). Based on that, it is necessary for SDEA and its bioactive compounds to screen the potential HDI before moving into clinical trials stage. Thus, a reliable HDI test assay is needed.

Compared to *in vivo* animal test, *in vitro* CYP450 probe substrate approach can provide reliable context and prospective knowledge in terms of less cost and time (Bjornsson et al., 2003; Venkatakrishnan et al., 2003), which are widely used in DDI and HDI study. Moreover, since human and animals (rat, mice et al.) have essential differences in basic tissue, cell structure and metabolic types, *in vitro* HDI assay usually uses commercial human liver microsomes (HLM) or human recombinant CYP450 enzymes (Lu and Di, 2020). Traditional CYP450 substrate method owns low efficiency because it only uses one probe substrate to test the activity of one metabolic enzyme at a time (“one-in-one” assay), which is difficult to meet the requirements of high throughput screening (Lin and Lu, 1998). However, as the development of high performance liquid chromatography-tandem mass spectrometry (HPLC-MS/MS), this problem has been solved finally. The triple quadrupole LC-MS can select specific substrate metabolites for determination by using multiple reaction monitoring (MRM), which has better selectivity and specificity compared with other analytical methods (liquid chromatography-UV, fluorescence and luminescence detections, *etc.*) (Youdim and Saunders, 2010). Based on the advantages of LC-MS/MS, the cocktail CYP450 assay (“N-in-one” assay) have been developed, which can simultaneously test the inhibitory effects of several CYP450 isoforms in one assay, with the effect of significantly reducing cost and time to evaluate DDI or HDI (Li et al., 2015; Liua et al., 2015; Spaggiari et al., 2016). The cocktail assay is very suitable for high-throughput screening in drug development process, especially for early drug metabolism studies, which is extremely important for dose design of those compounds with multiple metabolic pathways. It has been applied to monitor the *in vitro* inhibition activity of various CYP450 enzymes and time-dependent inhibition study (Chen et al., 2016). Therefore, in our study, an *in vitro* cocktail CYP450 assay was established with LC-MS/MS to reflect the activities of corresponding CYP450 isoforms. This assay was able to monitor the metabolic changes of specific substrates and validated by known enzymes inhibitors, so that it could achieve more accurate detection, better sensitivity and less interference.

In summary, we established an *in vitro* cocktail CYP450 assay by LC-MS/MS to detect the inhibitory effects of SDEA and its four constituents (Amentoflavone, Palmatine, Apigenin and Delicaflavone) on seven human CYP450 isoforms: CYP1A2, CYP2C8, CYP2C9, CYP2C19, CYP2D6, CYP2E1 and CYP3A. To determine whether SDEA and its components are the potential inhibitors of any of those important human CYP450 isoforms. Our research could reveal the metabolic interactions between CYP450 enzymes and SDEA constituents, which could clarify the clinical safety issues and promote the drug development of SDEA and its constituents.

## 2 Materials and methods

### 2.1 Materials and chemicals

Pooled mixed gender human liver microsomes from 50 donors were purchased from XenoTech (Lenexa, United States), β-nicotinamide adenine dinucleotide phosphate (NADPH) were purchased from solarbiobiotech (Beijing, China), Omeprazole (HPLC purity> 99%), Taxol (HPLC purity> 99%), Tolbutamide (HPLC purity> 99%), Chlorzoxazone (HPLC purity> 99%), Dextromethorphan Hydrobromide (HPLC purity> 98%), Alpha-Naphthoflavone (HPLC purity> 98%), Fluconazole (HPLC purity> 98%), Quercetin (HPLC purity> 98%) and Ketoconazole (HPLC purity> 99%) were purchased from Dalian Meilunbio. Co. Ltd (Dalian, China), Phenaceti (HPLC purity> 99%), Sulfaphenazolum (HPLC purity> 99%), Quinidine (HPLC purity> 99%) were purchased from Shyuanye Biotechnology Co. Ltd (Shanghai, China), Testosterone (HPLC purity> 98%) were purchased from Derick Biotechnology Co. Ltd (Chengdu, China), 4-Methylpyrazole (HPLC purity> 97%) were purchased from J&K Scientific (San Jose, United States).

The SDEA extract was prepared following our previously described procedure (Sui et al., 2016; Yao et al., 2017). Delicaflavone (purity≥ 98%, determined by the peak area normalization method using HPLC-PDA) were isolated from *S. doederleinii* and the structure was fully elucidated by MS, UV, ^1^H-NMR and ^13^C-NMR, which was confirmed by comparison with the literatures (Li et al., 2013; Li et al., 2014; Yao et al., 2017; Chen et al., 2018). Amentoflavone (HPLC purity> 98%) and Apigenin (HPLC purity> 98%) were purchased from Dalian Meilunbio. Co. Ltd (Dalian, China), Palmatine (HPLC purity> 98%) was purchased from Shyuanye Biotechnology Co. Ltd (Shanghai, China).

Methanol and acetonitrile (HPLC grade) were purchased from Merck KGaA (Darmstadt, Germany), formic acid (HPLC grade) was purchased from Aladdin (Shanghai, China), ethanol (analytical grade) was obtained from Sinopharm Chemical Reagents (Shanghai, China), and ultrapure water was prepared by a Millpore Milli-Q system (Beddford, United States).

### 2.2 Quantitative analysis of four constituents in SDEA

Amentoflavone, Delicaflavone, Apigenin, Palmatine and Rutin (internal standard) were precisely weighed and dissolved in methanol to obtain a stock solution with a concentration of 1 mg/ml, which was stored in refrigerator at 4°C. Before use, the stock solution was diluted with methanol into standard working solution and quality control working solution.

The concentrations of Amentoflavone and Apigenin standard curve working solution were: 3.125, 6.25, 12.5, 25, 50, 100, 200, 400 and 800 ng/ml; the concentrations of Amentoflavone and Apigenin quality control (QC) working solution were as 30, 150 and 650 ng/ml. The concentrations of Palmatine standard curve working solution were: 0.39, 0.78, 1.56, 3.125, 6.25, 12.5, 25, 50, 100 and 200 ng/ml; three level QCs were as 3, 30, and 150 ng/ml. The concentrations of Delicaflavone standard curve working solution were: 3.125, 6.25, 12.5, 25, 50, 100 and 200 ng/ml; and three QCs were as 8, 30 and 150 ng/ml. SDEA solid was accurately weighed and dissolved with methanol, then sonicated until complete dissolution. Diluted it with methanol to 1, 5, 25, 40 μg/ml and mix with the 40 ng/ml internal standard (Rutin) respectively. The mixture was vortexed for 2 min, then centrifuged at 13000 rpm for 10 min at room temperature. The supernatant was acquired for LC-MS/MS quantitative analysis.

### 2.3 Establishment of cytochrome P450 cocktail inhibition assay

Potassium phosphate buffer (200 µl, 0.1 M, pH 7.4) containing 1 mM NADPH, 0.5 mg/mL human liver microsomes, and a cocktail assay of seven probe substrates (Phenacetin for CYP1A2, Paclitaxel for CYP2C8, Tolbutamide for CYP2C9, Omeprazole for CYP2C19, Dextromethorphan for CYP2D6, Chlorzoxazone for CYP2E1, Dextromethorphan and Testosterone for CYP3A) or a single substrate (≤Km) were incubated at 37°C for 15 min (Supplementary Table S1). The contents of organic solvent and DMSO in incubation mixture was under 1% (v/v) and 0.1% (v/v) respectively. Reactions were terminated by adding 200 µL of an ice-cold stop solution consisting of methanol containing Rutin (2 µg/ml) as internal standard. Samples were subsequently cooled in ice bath to precipitate proteins. Supernatants were collected into clean tubes after centrifugation at 12000 rpm at 4°C for 10 min prior to inject into LC-MS/MS.

#### 2.3.1 Determination of linearity of metabolite formation

To determine the optimal incubation time for each CYP substrate, human liver microsomes (0.5 mg/mL) were incubated at 37°C for 0, 5, 10, 20, 30 and 60 min with each CYP substrates. All substrates concentration were 1 µM. After quantitative analysis using LC-MS/MS, the linearity of metabolite formation was evaluated.

#### 2.3.2 Validation of direct CYP450 inhibition

For the determination of inhibition curves using single substrate and the cocktail, seven selective CYP inhibitors were used at different concentrations as follows: 0.05–1 µM α-Naphthoflavone for CYP1A2, 1–50 μM Fluconazole for CYP2C19, 0.1–15 µM Quercetin for CYP2C8, 0.01–5 µM Sulfaphenazole for CYP2C9, 0.005–2 µM Quinidine for CYP2D6, 0.1–5 µM 4-Methylpyrazole for CYP2E1, and 0.005–1 µM Ketoconazole for CYP3A (Supplementary Table S2).

### 2.4 Concentration-dependent inhibitory effect of SDEA on CYP450 isoforms

Inhibitory effect of SDEA on CYP450 isoforms was determined by cocktail assay. SDEA at 10 concentrations from 0 to 200 µg/ml were used to measure IC_50_ value. The amount of metabolites produced by incubation without SDEA (control group) was taken as 100%, then draw inhibition efficiency curve.

### 2.5 Concentration-dependent inhibitory effect of four components in SDEA on CYP450 isoforms

Single-point inhibition was assessed by Delicaflavone, Amentoflavone, Apigenin and Palmatine at 10 µM, using the same protocol as described in Section 2.3. Percentages of control activity were calculated to determine inhibition potency. The inhibition curves were obtained by incubating the cocktail of substrates with 10–11 different compounds’ concentrations, where Delicaflavone and Amentoflavone were 0–50 µM.

### 2.6 Time-dependent inhibition study of SDEA, Delicaflavone and Amentoflavone

In order to explore whether SDEA, Delicaflavone and Amentoflavone have time-dependent inhibition on CYP450 isoforms, the cocktail inhibition assay (Section 2.3.) was used. First, SDEA (0.5 µg/ml), Delicaflavone (2 µM), and Amentoflavone (0.1 µM) were incubated with HLM (0.5 mg/ml) in the presence and absence of 1 mM NADPH for 35 min. After pre-incubation, a 20 µl aliquot of the incubation mixture was transferred to 180 µl potassium phosphate buffer (0.1 M, pH 7.4) containing 1 mM NADPH and mixed probe substrates for secondary culture, then stopped reaction after 15 min. Enzymes that may be involved in time-dependent were pre-incubated for 0, 5, 10, 15 and 35 min respectively, and then re-incubated to determine the type of inhibition. Residual enzyme activity pre-incubated without NADPH was set as the control group, then drawing suppression curves based on the measured data.

### 2.7 LC-MS/MS conditions

All metabolites and surrogate standards were analyzed by using LC-MS/MS on a Shimadzu (Kyoto, Japan) LCMS-8040 triple quadrupole mass spectrometer. Chromatographic separation was performed using an Ultimate® XB-C18 (100 × 2.1 mm, 3 μm) with gradient elution of water (0.2% formic acid) and methanol at 0.3 mL/min (gradient B). Column maintained at 40°C. 5 μl of supernatant was injected for LC-MS/MS analysis.

The following optimized MS parameters were used: ion spray voltage: 6.0 kV; Nebulizer gas flow: 3 L/min; Drying gas flow: 12 L/min; DL temperature: 250°C; Heatblock temperature: 400°C.

#### 2.7.1 Quantitative analysis of SDEA constituents

The gradient elution procedure is: 0–1 min, 30%–45% B; 1–3 min, 40%–75% B; 3–5 min, 75%–85% B; 5–8 min, 85%–95% B; 8–12 min, 95% B; 12–13 min, 95%–30% B; 30% B for equilibration.

Detection was carried out using electrospray with polarity switching, collision-induced dissociation, and multiple reaction monitoring (MRM). The MRM mode transition: *m/z* 536.90 → 375.00 (−) for Amentoflavone; *m/z* 538.9 → 256.95 (+) for Delicaflavone; *m/z* 352.00 → 336.10 (+) for Palmatine; *m/z* 271.15 → 153.05 (+) for Apigenin; *m/z* 611.00 → 302.85 (+) for Rutin (IS). The collision energies (CE) of the four components were 33 V, −45 V, −35 V, −35 V and −35 V respectively.

#### 2.7.2 Determination of the inhibitory effects of SDEA and four constituents on CYP450 enzymes by cocktail assay

A gradient condition was applied with the following program: 0–1 min, 10%–20% B; 1–1.01 min, 20%–75% B; 1.01–3 min, 75%–77% B; 3–5 min, 77%–80% B; 5–8 min, 80%–85% B; 8–9 min, 85%–95% B; 9–12 min, 95% B; and then 10% B for equilibration. For specific MRM parameters, please refer to Supplementary Table S3.

### 2.8 Data analysis

Quantitative LC-MS/MS data were analyzed using Shimadzu LabSolutions software (Kyoto, Japan). The Km and IC_50_ values were determined by using the GraphPad Prism 5.0 software (San Diego, United States).

## 3 Results

### 3.1 The content of four constituents in SDEA

**FIGURE 1:**
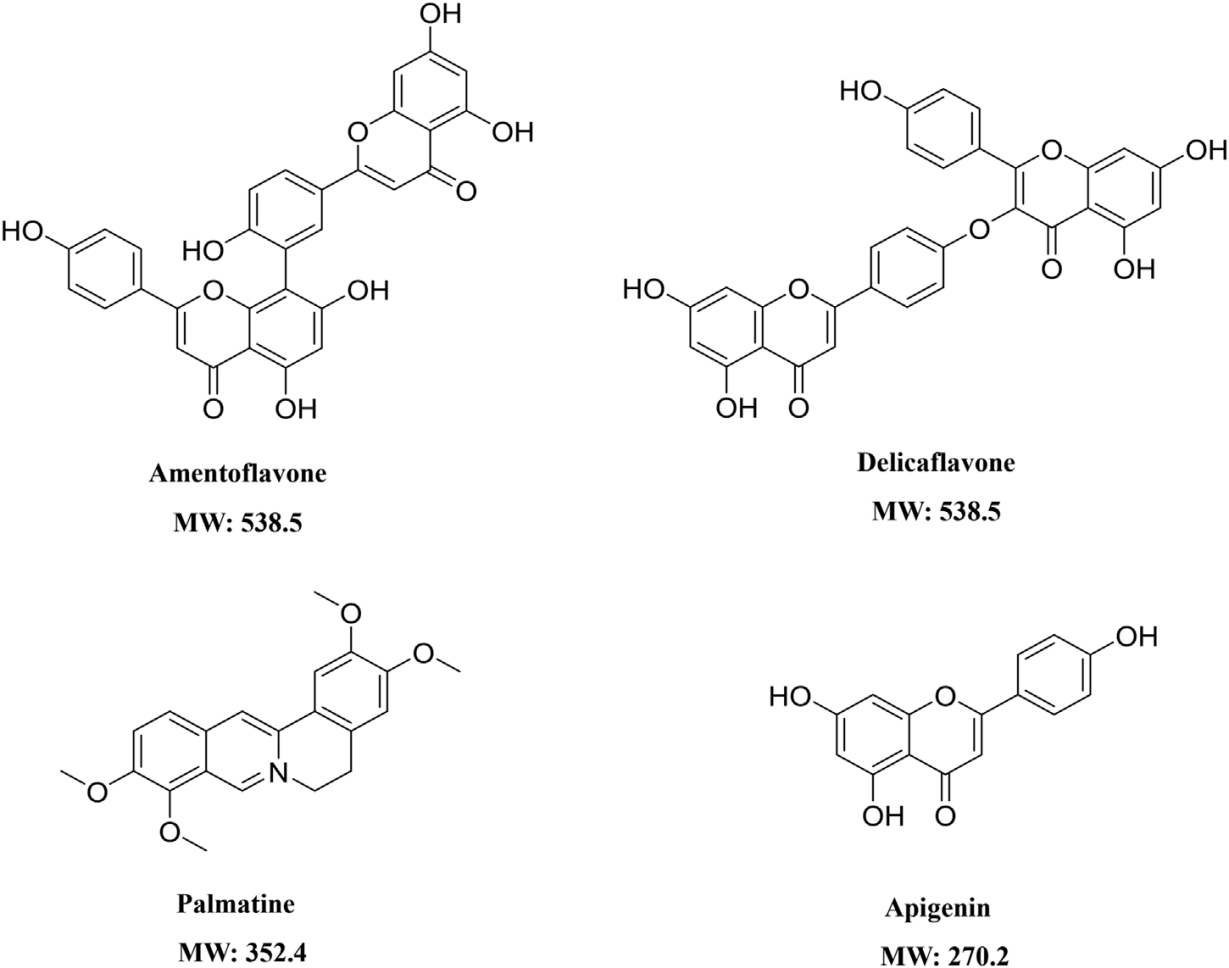
Structural formula and molecular weight of the components in SDEA.

The structures of Amentoflavone, Delicaflavone, Palmatine, and Apigenin were shown in Figure 1. Amentoflavone and Apigenin were linearly related in a range of 3.125–800 ng/mL by using 1/X^2^ weighting. Palmatine showed a linear relationship in the range of 0.391–200 ng/ml, and Delicaflavone showed a linear relationship in the range of 3.125–200 ng/ml. The four SDEA constituents showed a good linear relationship over their concentration range with coefficient of determination *r* ^2^ ≥ 0.99 (Supplementary Table S4).

QC samples in three levels (Low QC, Medium QC and High QC) were prepared separately and used to evaluate those quantitative methods, all samples were analyzed by three replicates. The related standard deviation (RSD) of the tested compounds ranged from −8.88% to 12.60% (Supplementary Table S4). MRM mass spectrum of blank sample and Limit of Quantity (LOQ) were displayed in Supplementary Figure S1. As well, all of them demonstrated the feasibility of this analytical methods.

1 μg/ml, 5 μg/ml, 25 μg/ml and 40 μg/ml SDEA samples were prepared and injected into LC-MS/MS respectively. Using the established quantitative method, the content of four constituents in SDEA was determined. The average w/w % (Weight compound/weight extract) showed that Delicaflavone, Palmatine, Apigenin were 2.21%, 0.019% and 0.024% respectively, and Amentoflavone reached 13.65%, which was the most abundant one in those four constituents (Table 1).

**TABLE 1:** Contents (% w/w) of the four constituents in SDEA. (Higher SDEA concentrations were used because Apigenin and Palmatine are less in SDEA).

Compound	SDEA conc. (μg/ml)	Content (%)	Average content (%)
		% weight compound/weight extract	
Amentoflavone	1	13.78	13.65
	5	13.53	
Delicaflavone	1	2.29	2.21
	5	2.13	
Palmatine	5	0.019	0.019
	25	0.019	
Apigenin	25	0.026	0.024
	40	0.023	

### 3.2 Development of CYP450 cocktail assay

The probe substrates in this cocktail assay were recommended based on FDA guidance. CYP3A sub-family can bind multiple structurally different substrates (Wrighton et al., 2000; Ekroos and SjOgren, 2006; Watanabe et al., 2007), it is necessary to use more than two CYP3A *in vitro* marker reactions to evaluate the activity of CYP3A (Wrighton et al., 2000), so that can show more accurate results (Galetin et al., 2005). We selected Testosterone and Dextromethorphan as two different substrates of CYP3A subfamily, their specific products metabolized by CYP3A were Testosterone: 6β-hydroxytestosterone; Dextromethorphan: 3-methoxmorphine. The MRM parameters of substrates corresponding to seven human CYP450 isoforms were summarized in Supplementary Table S3. LC-MS/MS chromatography of substrates and their metabolites were included in Supplementary Figure S2.

To determine the optimist incubation time for all substrates, we measured time-dependent trends in amount of metabolites produced by seven CYP450 enzyme substrates. Although Dextromethorphan (CYP2D6), Chlorzoxazone (CYP2E1), Paclitaxel (CYP2C8) remained linear for first 20 min and metabolic rates of Tolbutamide (CYP2C9), Omeprazole (CYP2C19), Phenacetine (CYP1A2) were linear for up 15 min, Testosterone (CYP3A4) remained linear only for first 10 min and then flattened out. Trend diagram of the generation of metabolites for seven enzyme substrates with time was included in Supplementary Figure S3. Compromising sensitivity to detect metabolites formed at low substrate concentrations and high inhibitor concentrations, an incubation time of 15 min was finally determined.

Considering the linear curve of metabolic reaction, the final concentration of substrate is usually lower than its Km value in order to ensure linear relationship of metabolic rate range and high affinity. What’s moreover, enzyme-substrate affinity data illustrated that the Km value should be similar for a specific enzyme and substrate, regardless of enzyme source (Donnell et al., 2007). Since the incubated substrates could interact with each other (Spaggiari et al., 2014), reducing substrate concentration is the most direct and effective way to reduce the interference (Pillai et al., 2013). Therefore, the final incubation concentration of each substrate was ideal when it was below Km. According to the recommended data in literature (Spaggiari et al., 2014) and sensitivity of MS, final substrate concentration in assay were determined: Phenacetin (1A2), Paclitaxel (2C8) and Tolbutamide (2C9) were 10 μM, Dextromethorphan (2D6) and Testosterone (3A) were 5 μM, Omeprazole (2C19) was 2 μM, and Chlorzoxazone (2E1) was 15 μM. All data were summarized in Supplementary Table S1.

**FIGURE 2:**
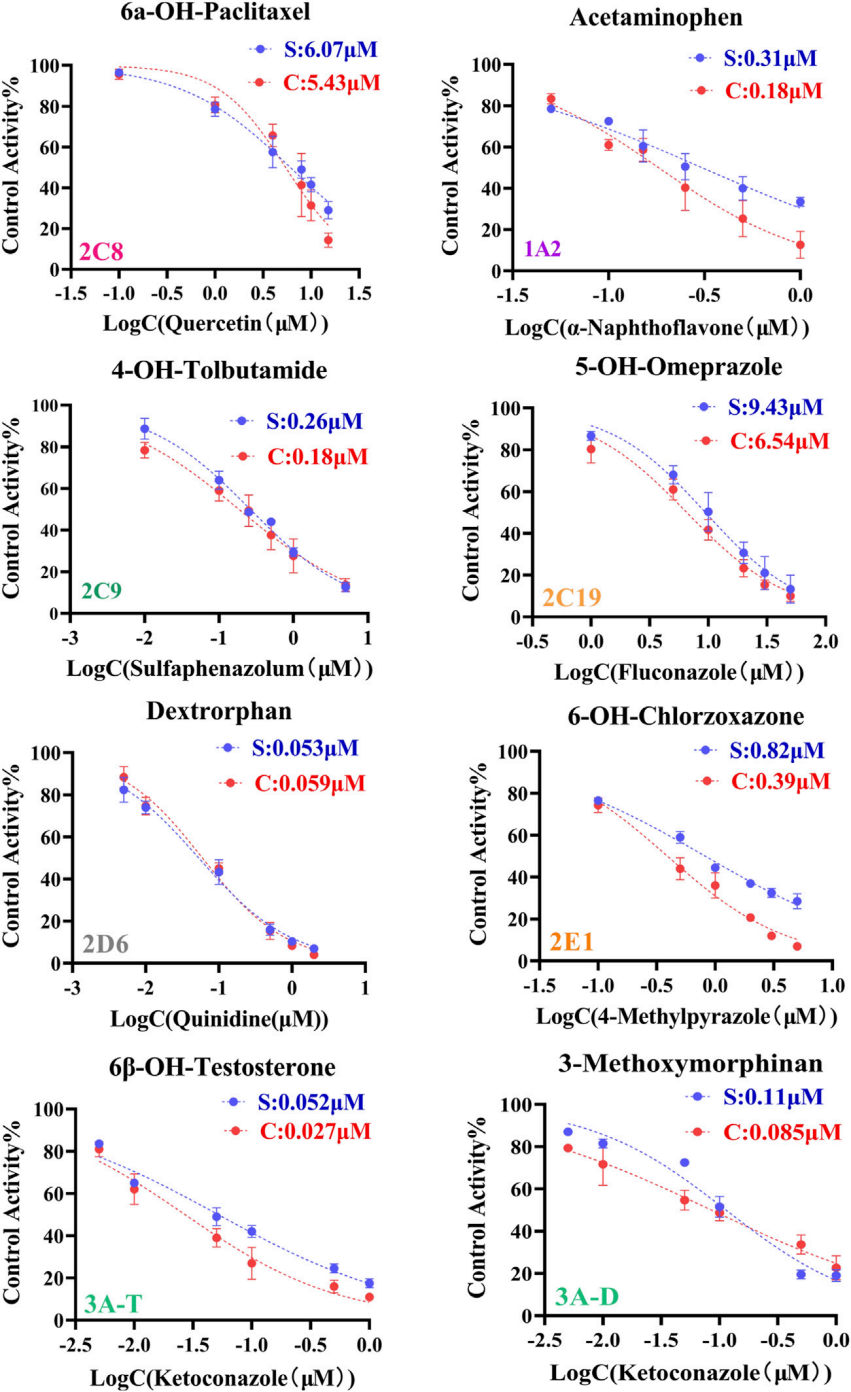
Inhibition curves each substrate incubated with cocktail assay and single substrate method. Note: 3A-T, CYP3A (Testosterone); 3A-D, CYP3A (Dextromethorphan).

The IC_50_ values obtained by cocktail assay was compared with a single substrate using known inhibitors of each enzyme reaction alone, so as to verify the feasibility of this assay. a-Naphthoflavone was inhibitor of CYP1A2, our cocktail assay showed that IC_50_ value of a-Naphthoflavone on CYP1A2 was 0.18 µM, and the value was 0.31 µM from single substrate assay, which indicated similar results. The IC_50_ value of Quercetin on CYP2C8 was 5.43 µM in cocktail assay, while the number was 6.07 µM in single substrate experiment, all of them were located in desirable range by literature (Kozakai et al., 2012; Chen et al., 2016; Valicherla et al., 2019). IC_50_ values of other CYP450 isoforms inhibitors (Sulfaphenazole for CYP2C9, Fluconazole for CYP2C19, Quinidine for CYP2D6, 4-Methylpyrazole for CYP2E1, Ketoconazole for CYP3A) on cocktail assay and single substrate method were also performed and compared. Inhibition curves were shown in Figure 2 and IC_50_ values were summarized in Supplementary Table S2, all of them showed good agreement between cocktail assay and single-substrate approach, which indicated the accuracy of this cocktail inhibition assay.

### 3.3 Concentration-dependent inhibition effect of SDEA and four constituents on CYP450 isoforms

**FIGURE 3:**
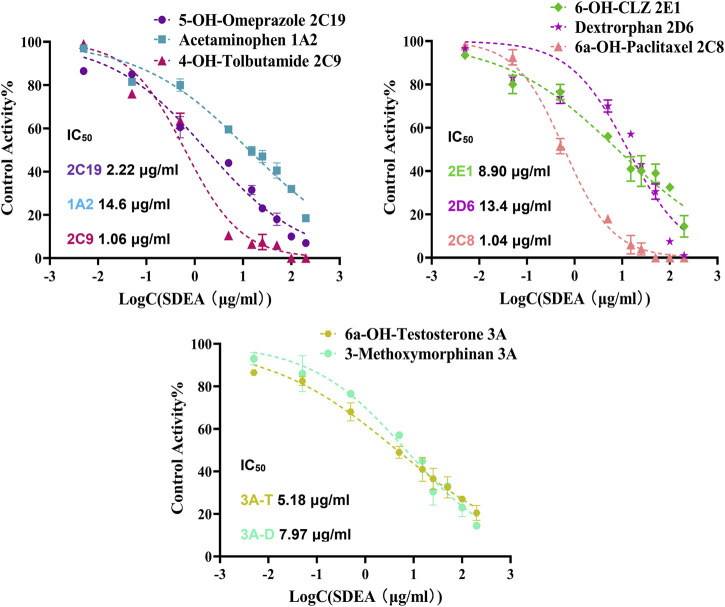
Inhibitory potency curves and IC_50_ values of SDEA on seven CYP450 enzyme isoforms. Note: 3A-T, CYP3A (Testosterone); 3A-D, CYP3A (Dextromethorphan).

The established cocktail assay was used to determine potential HDI of SDEA. Results showed that SDEA has strong inhibitory effect on CYP2C8 and CYP2C9, where IC_50_ values were 1.04 μg/ml and 1.06 μg/ml. SDEA also showed moderate inhibitory ability on CYP2C19 (IC_50_ was 2.22 μg/ml), CYP2E1 (IC_50_ was 8.90 μg/ml) and CYP3A (IC_50_ was 5.18 μg/ml for Testosterone and 7.97 μg/ml for Dextromethorphan). The inhibition activities for CYP1A2, CYP2D6 were mildly, both IC_50_ values were above 10 μg/ml (Figure 3).

**FIGURE 4:**
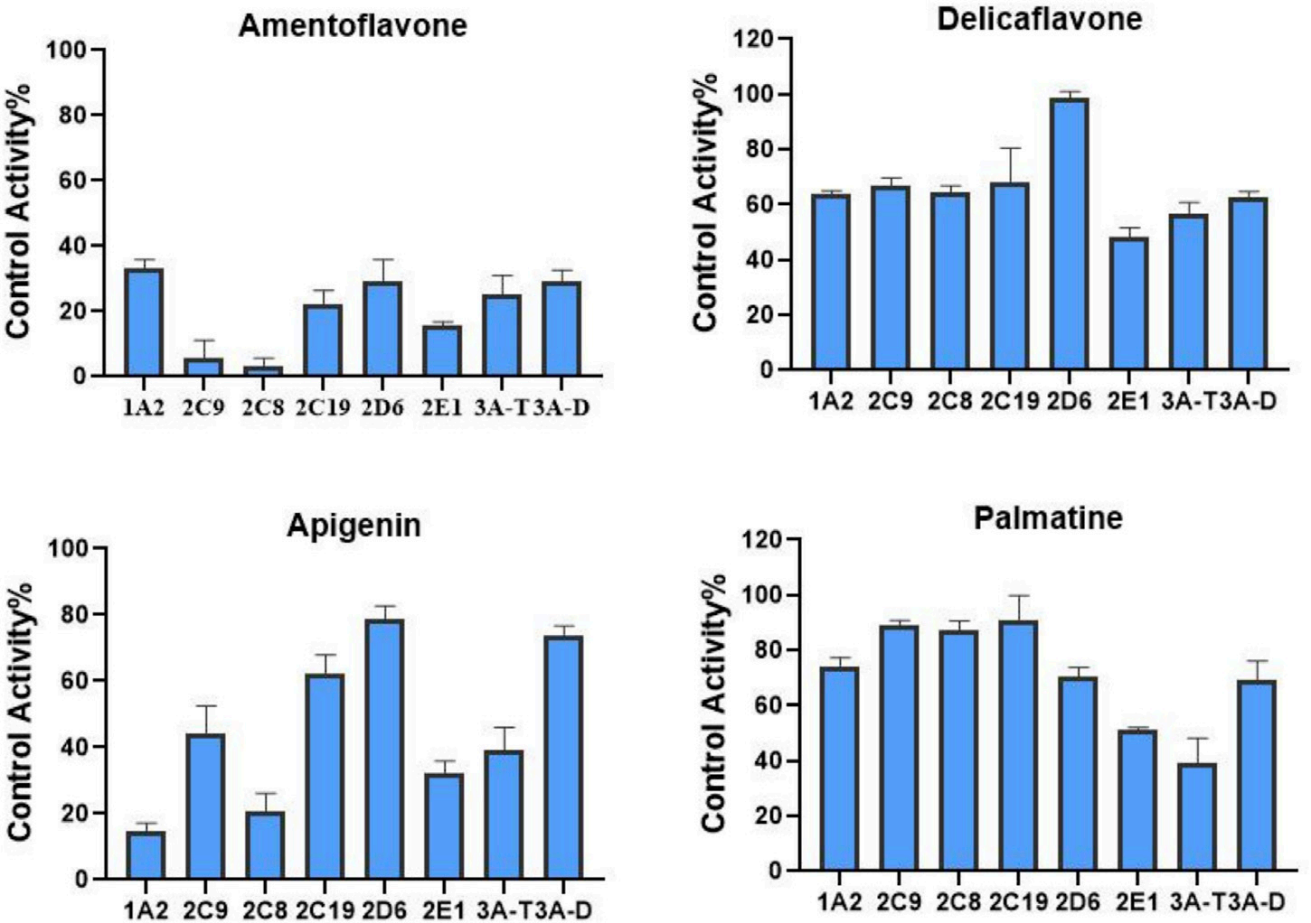
Inhibition of CYP enzymes by Amentoflavone, Delicaflavone, Apigenin, and Palmatine at 10 μM. Inhibition Activity was expressed as a percentage of remaining activity compared to a control test without enzyme inhibitor. Note: 3A-T, CYP3A (Testosterone); 3A-D, CYP3A (Dextromethorphan).

We also determined the inhibitory effects of SDEA constituents on seven cytochrome P450 isoforms. At the concentration of 10 μM, Amentoflavone showed more than 50% inhibitory effect on seven enzyme types, the inhibitory effect of Apigenin on CYP1A2, CYP2C8, CYP2C9, CYP2E1 and CYP3A (Testosterone) was more than 50%. Palmatine inhibits CYP3A (Testosterone) by more than 50% and has a weak inhibitory effect on CYP2E1. Delicaflavone has a weak inhibitory effect on CYP2E1 and CYP3A, but it had no obvious inhibitory effect on the other CYP450 isomers at this concentration (Figure 4).

**FIGURE 5:**
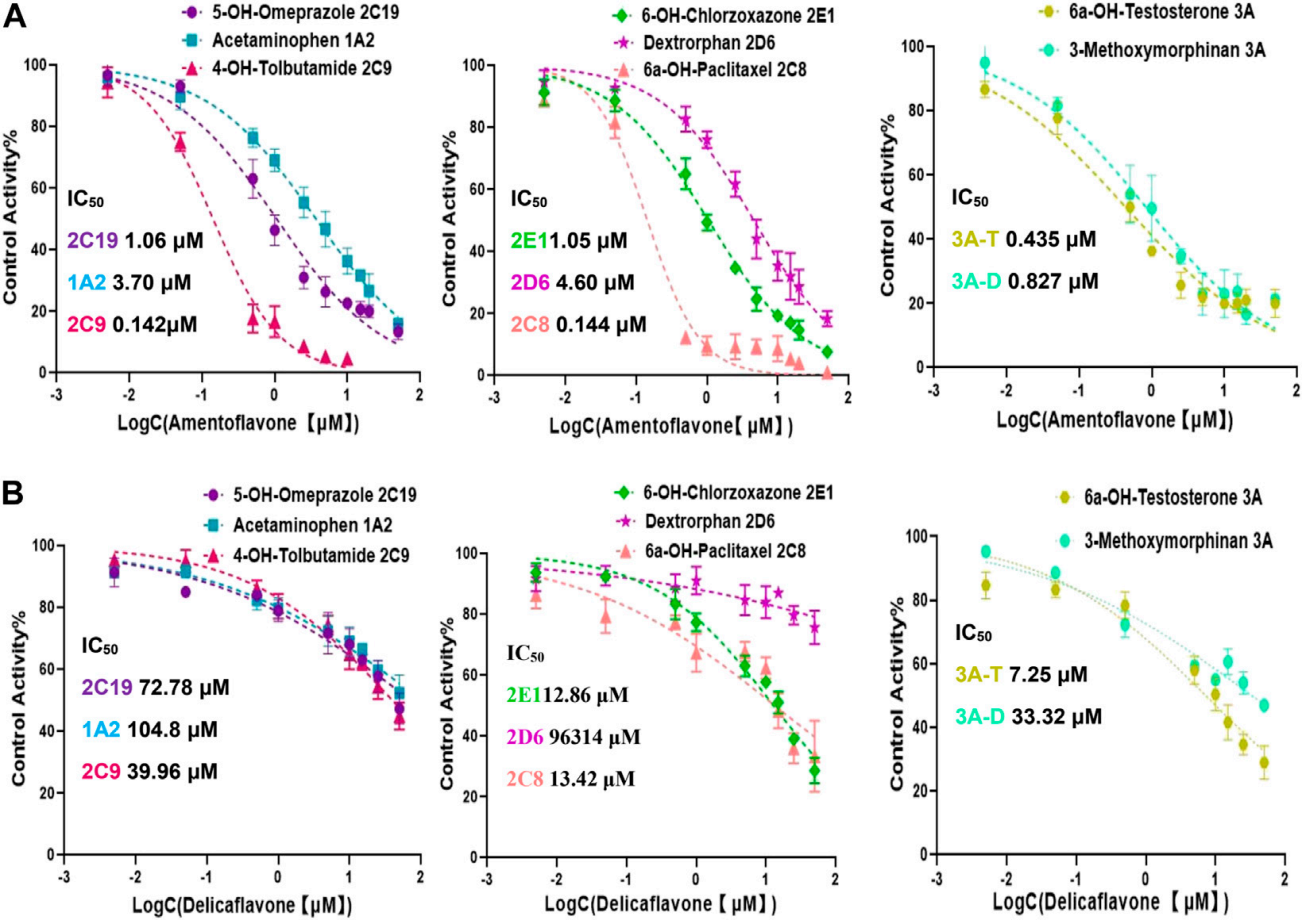
Inhibition curves and IC_50_ values of Amentoflavone **(A)** and Delicaflavone **(B)** against seven CYP450 isoforms. Note: 3A-T, CYP3A (Testosterone); 3A-D, CYP3A (Dextromethorphan).

Amentoflavone were most abundant in four SDEA constituents (13.65% w/w, Table 1), which showed a broad-spectrum potent CYP enzymes inhibitory effect. The IC_50_ values were less than 5 μM for the seven cytochrome P450 isoforms, especially for CYP2C9, CYP2C8 and CYP3A, the IC_50_ values were even less than 0.5 μM (Figure 5A). In contrast, Delicaflavone only had a moderate inhibitory effect on CYP3A (Testosterone), weak inhibitory effect on CYP2C8, CYP2E1 and had almost no inhibitory effect on the other CYP isoforms (Figure 5B).

### 3.4 Time-dependent inhibition study

**FIGURE 6:**
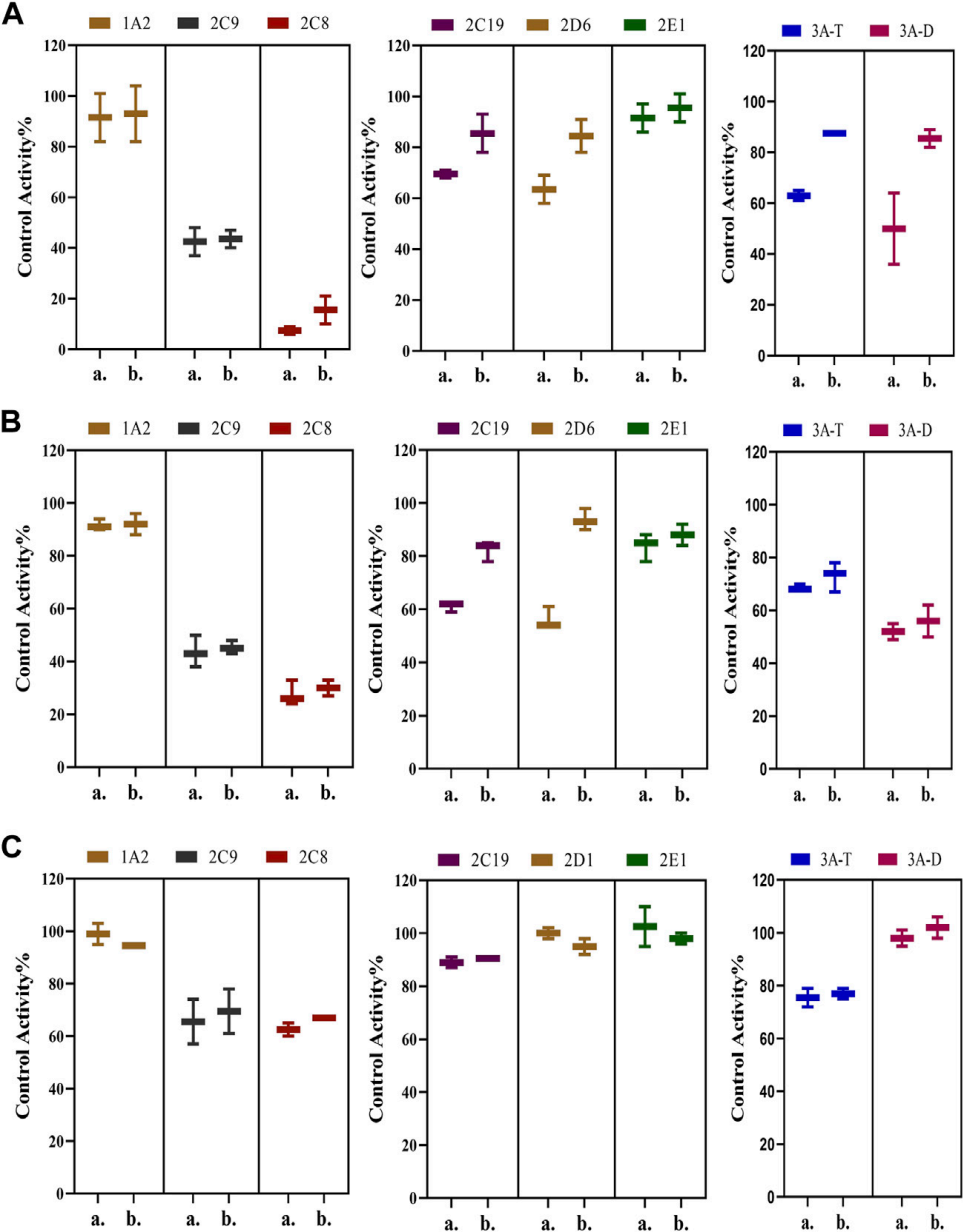
Percentage reduction in activity of seven enzymes after incubation with SDEA **(A)**, Amentoflavone **(B)** and Delicaflavone **(C)** using a single point time-dependent inhibition screening assay. The experiment was carried out three times. Note: (1) a. presence of NADPH; b. without NADPH. (2) 3A-T, CYP3A (Testosterone); 3A-D, CYP3A (Dextromethorphan).

Possible time-dependent inhibition of SDEA, Amentoflavone and Delicaflavone was tested using cocktail assay. Single point screening assay showed that SDEA (0.5 μg/ml) demonstrated a time-dependent inhibition on CYP2C19, CYP2D6, CYP3A (Testosterone) (Figure 6A), and Amentoflavone (0.1 μM) showed the time-dependent inhibition on CYP2C19, CYP2D6 (Figure 6B). While Delicaflavone (2 μM) did not produce time-dependent inhibition effect on those enzymes (Figure 6C)**.**

**FIGURE 7:**
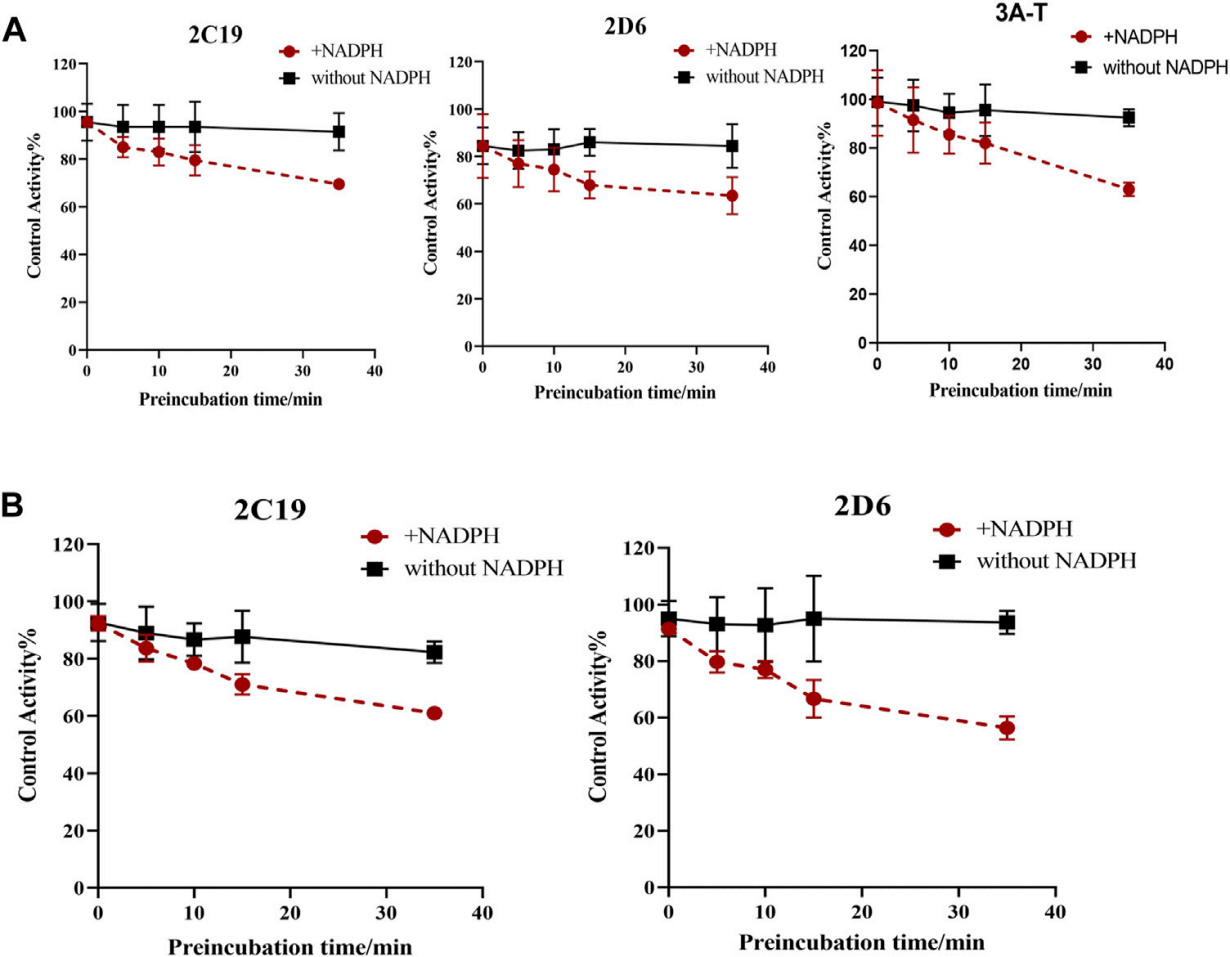
Effect of incubation time on the inhibition of CYP3A, CYP2D6 and CYP2C19, with SDEA **(A)** and Amentoflavone **(B)**. Note: 3A-T, CYP3A (Testosterone).

Based on these results, we have determined the inhibition effect of incubation time with SDEA or Amentoflavone on CYP2C19, CYP2D6, CYP3A (Testosterone) activity. When HLM was co-incubated with SDEA (0.5 μg/ml) in presence of NADPH for 0–35 min, the metabolic activity of CYP2C19 decreased from 95.5% to 69.5%; the activity of CYP2D6 decreased from 84.5% to 63.5% and CYP3A (Testosterone) decreased from 98.5% to 63% (Figure 7A). As for Amentoflavone (0.1 μM), the CYP2C19 activity decreased from 92% to 61% and CYP2D6 activity decreased from 91% to 56%, during the 0–35 min of incubation with HLM and NADPH (Figure 7B). The control group was without NADPH, after 0–35 min co-incubation of HLM with SDEA or Amentoflavone, no significant change in enzymes activity was observed. Those data indicated that SDEA and Amentoflavone may follow a time-dependent inhibition on CYP450 enzymes.

## 4 Discussion

*In vitro* model of human CYP450 enzymes inhibition cocktail assay used in this study can discover the potential of interaction between herb and drugs, reduce the risk of using clinical drug, lay a foundation for clinical medication. Incubation time (15 min) and substrate concentration were determined by combining literature data with experimental conditions (Supplementary Table S1). The established cocktail assay was then validated with known CYP450 enzymes inhibitors, and was applied to evaluate whether SDEA and its bioactive constituents produce concentration-dependent or time-dependent inhibition on the selected CYP450 enzymes. Amentoflavone and Delicaflavone are the main active pharmacological components of SDEA and have high content in SDEA. Palmatine and Apigenin are also SDEA constituents and have been reported to show inhibitory effects on CYP450 enzymes (von Moltke et al., 2004; Kim et al., 2000). Moreover, Palmatine and Apigenin are more common in everyday life than other ingredients, the results will be more practical. Thus, those four compounds were selected for HDI test.

The experiment results showed that SDEA had a strong concentration-dependent inhibition effect on CYP2C9 and CYP2C8 (Figure 3), time-dependent inhibition effect on CYP2D6, CYP2C19 and CYP3A (Figure 7A), which may lead to some serious adverse effects in clinical use. CYP3A subfamily is a particularly important CYP metabolizing, mediating over 50% *in vivo* biotransformation of clinical drugs (Liu et al., 2006; Xia et al., 2015). In addition, CYP3A subfamily also plays an important role in maintaining metabolic balance of important endogenous substances such as bile acids, steroid hormones and cholesterol (Liang et al., 2015; Xia et al., 2015; Liang et al., 2016), and it is also the main metabolic enzyme of many narrow-window drugs, such as Paclitaxel, Bortezomib, and Gefitinib. SDEA not only inhibits CYP3A (IC_50_ < 10 μg/ml), more importantly, after 35 min co-incubation of SDEA with NADPH, CYP3A activity was reduced from 98.5% to 63%, which significantly increases the risk of herb-drug interaction with serious consequences. Some oral hypoglycemic agents, such as Glyburide, Rosiglitazone, and Repaglinide, are mainly metabolized by CYP2C8 or CYP2C9, it may increase hypoglycemia risk when those medicines are used together with SDEA. For epilepsy patients, SDEA should be used with caution when taking Phenytoin and Valproic acid (metabolized by CYP2C19) at the same time, in order to avoid serious adverse reactions.

The contents of Amentoflavone and Delicaflavone in SDEA were 13.65% w/w and 2.21% w/w respectively, which were similar to results of previous studies (Li et al., 2013) (Table 1). Amentoflavone not only shows pharmacological activities, such as anti-tumor (Ndongoa et al., 2015), antiviral (Coulerie et al., 2013), antioxidant (Arwa et al., 2015), anti-inflammatory (Abdallah et al., 2015), but also has therapeutic effects on central nervous (Zhang et al., 2015) and cardiovascular system (Yu et al., 2017). The data indicated that Amentoflavone may be the main contributor for SDEA to inhibit CYP enzymes activity. Amentoflavone showed high content in SDEA and strong or moderate inhibitory effect on CYP1A2, CYP2C8, CYP2C9, CYP2C19, CYP2D6, CYP2E1, CYP3A (Figure 5A). The inhibitory effect was even stronger than some specific inhibitors of CYP2C9, CYP2C8 and CYP3A. In previous reports, Amentoflavone had a strong inhibitory effect on various CYP450 isoforms such as CYP2C9, CYP2C19, CYP2D6 and CYP3A4 (von Moltke et al., 2004; Kimura et al., 2010), which were consistent with the experiment results. It should be noted that this paper found a strong inhibitory effect on CYP2C8 and a moderate inhibitory effect on CYP1A2 and CYP2E1 by Amentoflavone, which were not reported in previous article (von Moltke et al., 2004). The researchers did not find the inhibitory effect of Amentoflavone on CYP1A2, which may be related to the differences in enzyme sources, substrate types and concentrations.

Similar to SDEA, Amentoflavone also has time-dependent inhibitory effects on CYP2D6 and CYP2C19 (Figure 7B). Although CYP2D6 accounts for a small fraction of CYP450 expression in liver, it participates in metabolism of various drugs. As a therapeutic drug for breast cancer, Tamoxifen (TAM) has better pharmacological activity only after the formation of 4-hydroxy-N-demethylamoxifen (Endoxifen), which is a metabolite catalyzed by CYP2D6 (Kleina et al., 2013). What’s more, CYP2D6 is the most polymorphic metabolic enzyme, which is of great significance in genetics, especially in pharmacogenetics. CYP2D6 and CYP2C19 are jointly involved in the metabolism of psychotropic drugs, such as Selective Serotonin Recycle Inhibitors (SSRIs), Clozapine (CZ) (Caetano1 and Piatkov, 2016; Menkes et al., 2018) and Risperidone. For those patients who have weak CYP2D6 and CYP2C19 activities, the inhibition is more dangerous and may lead to serious consequences (Lymperopoulos et al., 2015). A recent study has found that Amentoflavone was a strong and broad-spectrum UDP-glucuronosyltransferase (UGT) inhibitor (Lv et al., 2018). Since UGT enzymes involve in the most phase II elimination in body, the inhibition of UGT would bring potential risks for those medicines mainly clearing by this pathway. Considering the relationship of SDEA and Amentoflavone, we speculate that SDEA may also have the potential to produce significant inhibition on UGT, which needs further experiments to confirm.

Because the content determination and single concentration inhibition showed that Palmatine and Apigenin contents were low (Table 1), and the inhibitory effect on enzyme type was not as strong as Amentoflavone (Figure 4), so no further time-dependent study was conducted for Palmatine and Apigenin. Unlike SDEA, Amentoflavone, Apigenin or Palmatine, Delicaflavone shown no significant inhibition effect on the selected CYP450 isomers at 10 μM, only a weak inhibitory effect on CYP2E1 and CYP3A. Although Delicaflavone is the isomer of Amentoflavone, the difference on CYP450 enzymes inhibition effect may account in the steric hindrance. There are active site (responsible for substrate biding and NADPH-CYP450 oxidase reaction) and allosteric site (responsible for outside molecules to modulate enzyme activity) in CYP450 enzymes (Deodhar et al., 2020). It is possible that Amentoflavone binds to the allosteric site on CYP450 enzymes and lead to a strong inhibition effect. Delicaflavone did not bind to or weakly work on the allosteric site on CYP450 enzymes, resulting in the different CYP450 inhibition activities for those two isomers. Delicaflavone also has excellent anti-cancer and tumor-suppressing ability, the less influence on the CYP450 enzymes activity will make it a better candidate for next step drug development than SDEA or Amentoflavone.

## 5 Conclusion

Through the established reliable cocktail assay, SDEA was found to have concentration-dependent inhibition on several CYP450 enzymes, and inhibited CYP2D6, CYP2C19, CYP3A in a time-dependent manner. The inhibition activity may be mainly due to the higher content of component: Amentoflavone (13.65%). Amentoflavone has inhibitory effects on all tested CYP450 enzymes, the inhibitory effects on CYP2C9, CYP2C8 and CYP3A were even greater than the corresponding specific inhibitors. Amentoflavone also has time-dependent inhibition on CYP2D6 and CYP2C19. The other two constituents from SDEA, Apigenin and Palmatine, both showed concentration-dependent inhibition. For Delicaflavone, no significant inhibitory effect on CYP450 enzymes was observed. Since Delicaflavone owns excellent anti-cancer ability and low HDI potential, it was more suitable to develop as a new anti-cancer drug.

## Data Availability

The original contributions presented in the study are included in the article/Supplementary Material, further inquiries can be directed to the corresponding author.
