# Supplementary material for: Inhibitory effect of Selaginella doederleinii hieron on human cytochrome P450
Source: Front Pharmacol. 2023 Feb 15;14:1108867. doi: 10.3389/fphar.2023.1108867 (PMC9975586; doi:10.3389/fphar.2023.1108867)

Supplementary information

Inhibitory Effect of Selaginella doederleinii Hieron on Human Cytochrome P450

Fei Lin [1,+], Xinhua Lin[1,+], Xuewen Wang [2], Guanghui Mei [1], Bing Chen[1], Hong Yao[1] and Lingyi Huang [1,*]

[+] These authors contributed equally to this work.

[1]Department of Pharmaceutical Analysis, School of Pharmacy, Fujian Medical University, Fuzhou, China
[2]Department of Preventive Medicine, School of Public Health, Fujian Medical University, Fuzhou, China

*** Correspondence:**

Lingyi Huang

lingyi.huang@fjmu.edu.cn

Supplementary TABEL S1

Potential drug-drug interaction, recommended concentration and final concentration of each CYP450 substrate (Spaggiari et al., 2014).

Substrate<br>（CYP isoform）	Potential interaction	Recommended concentration	Final concentration in assay (μM)
Phenacetin<br>(1A2)	Low affinity pathways:<br>2C9, 2C19,2E1<br>Weakly inhibit 2B6<br>Strongly inhibit 2C9 (>50%)	< 50 μM	10 μM
Paclitaxel<br>(2C8)	May participate in metabolic pathways:<br>2C9, 3A4, 2C19<br>Moderate 2A6 inhibition<br>(<40%).	< 10 μM	10 μM
Tolbutamide<br>(2C9)	May participate in metabolic pathways:<br>2C19, 2C8	< 200 μM	10 μM
Omeprazole<br>(2C19)	Low affinity pathways:<br>2C8, 2C9, 2A6<br>Weakly inhibit 2C8, 2D6,<br>Strongly inhibit 1A2, 2C9	< 40 μM	2 μM
Dextromethorphan<br>(2D6)	Low affinity pathways:<br>2C9	< 25 μM	5 μM
Chlorzoxazone<br>(2E1)	Low affinity pathways:<br>1A2, 2C8, C19, 2D6, 3A<br>Moderately inhibits 2A6<br>Strongly inhibits 1A2, 3A	< 90 μM	15 μM
Testosterone<br>(3A)	Low affinity pathways:<br>2C9, 2C19<br>Moderately inhibits 1A2<br>Weakly inhibits 2C19, 2C9	< 10 μM	5 μM

Supplementary TABEL S2

The IC_{50} values of known inhibitors using single substrate method, new cocktail assay and literature values (Spaggiari et al., 2014).

Substrate	Inhibitor	Inhibitor concentration	Single substrate IC_{50} (μM)	Cocktail IC_{50} (μM)	Literature IC_{50} (μM)
Phenacetin (1A2)	a-Naphthoflavone	0.05, 0.1, 0.15, 0.25, 0.5, 1	0.31	0.18	0.01-0.5
Paclitaxel (2C8)	Quercetin	0.1, 1, 4, 8, 10, 15	6.07	5.43	1.09-7
Tolbutamide (2C9)	Sulfaphenazole	0.01, 0.1, 0.25, 0.5, 1, 5	0.26	0.18	0.069-1.3
Omeprazole (2C19)	Fluconazole	1, 5,10, 20, 30, 50	9.43	6.54	0.6337-5.9
Dextromethorphan (2D6)	Quinidine	0.005, 0.01, 0.05, 0.1, 1, 2	0.053	0.059	0.015-0.27
Chlorzoxazone (2E1)	4-Methylpyrazole	0.1, 0.5, 1, 2, 3, 5	0.82	0.39	0.87-15
Testosterone (3A)	Ketoconazole	0.005, 0.01, 0.05, 0.1 ,0.5, 1	0.052	0.027	0.019-1.8
Dextromethorphan (3A)	Ketoconazole	0.005, 0.01, 0.05, 0.1, 0.5, 1	0.11	0.085	0.019-1.8

Supplementary TABLE S3

MRM transition and collision energy (CE) of the substrate metabolites in the CYP450 cocktail assays.

Enzyme	Substrate	Substrate metabolite	MRM transition of metabolites (polarity)	CE (V)
1A2	Phenacetin	Acetaminophen	152.00>110.00(+)	-15
2C8	Paclitaxel	6α-OH-PAC	870.00>286.00(+)	-17
2C9	Tolbutamide	4-OH-TOL	287.00>171.00(+)	-18
2C19	Omeprazole	5-OH-OME	362.00>214.00(+)	-10
2D6	Dextromethorphan	Dextrorphan	258.20>157.00(+)	-40
2E1	Chlorzoxazone	6-OH-CLZ	183.80>120.00(-)	20
3A	Testosterone	6β-OH-TES	305.00>269.00(+)	-15
3A	Dextrmethorphan	3-Methoxymorphinan	258.20>15.00(+)	-40

Supplementary TABLE S4

The linear range, LOQ, regression equation and QC results for the four SDEA constituents.

Analyte	Linear range (ng/mL)	LOQ (ng/mL)	Regression equation(w=1/x^2)	QC(n=3)	
				Conc.(ng/mL)	RSD (%)
Amentoflavone	3.125~800	3.125	y=0.285x+0.028(R^2=0.995)	30	12.04%
				150	9.96%
				650	-1.59%
Delicaflavone	3.125~200	3.125	y=0.100x+0.010(R^2=0.992)	8	-8.88%
				30	12.60%
				150	5.07%
Palmatine	0.391~200	0.391	y=9.044x+0.103(R^2=0.994)	3	4.89%
				30	9.20%
				150	-1.23%
Apigenin	3.125~800	3.125	y=0.231x+0.025(R^2=0.997)	30	9.78%
				150	6.00%
				650	5.08%

Supplementary FIGURE S1

LC-MS/MS spectrum of blank sample and LOQ for the four SDEA constituents.

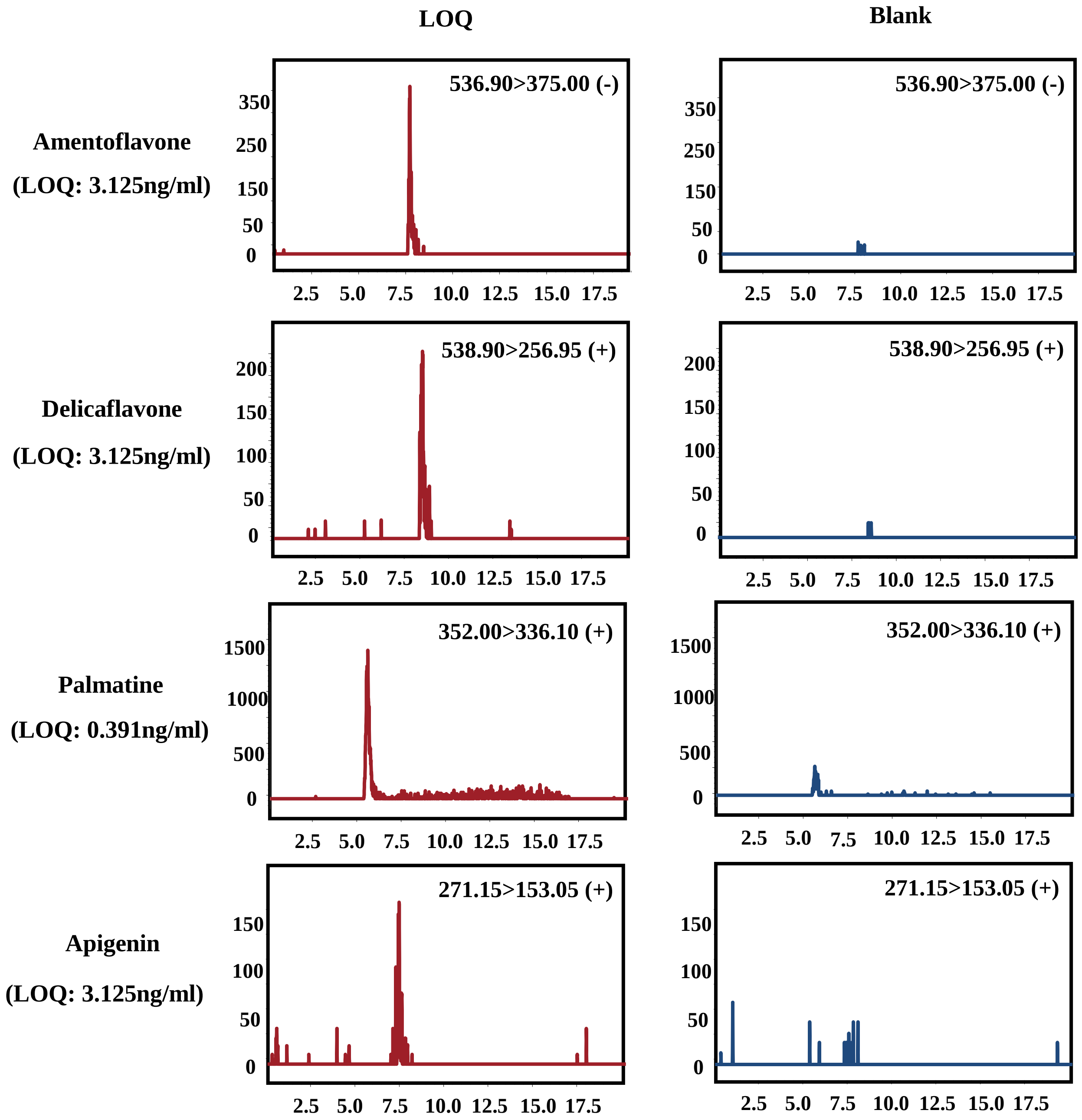

Supplementary FIGURE S2

MRM mass spectra of substrates, their specific metabolites and internal standard.

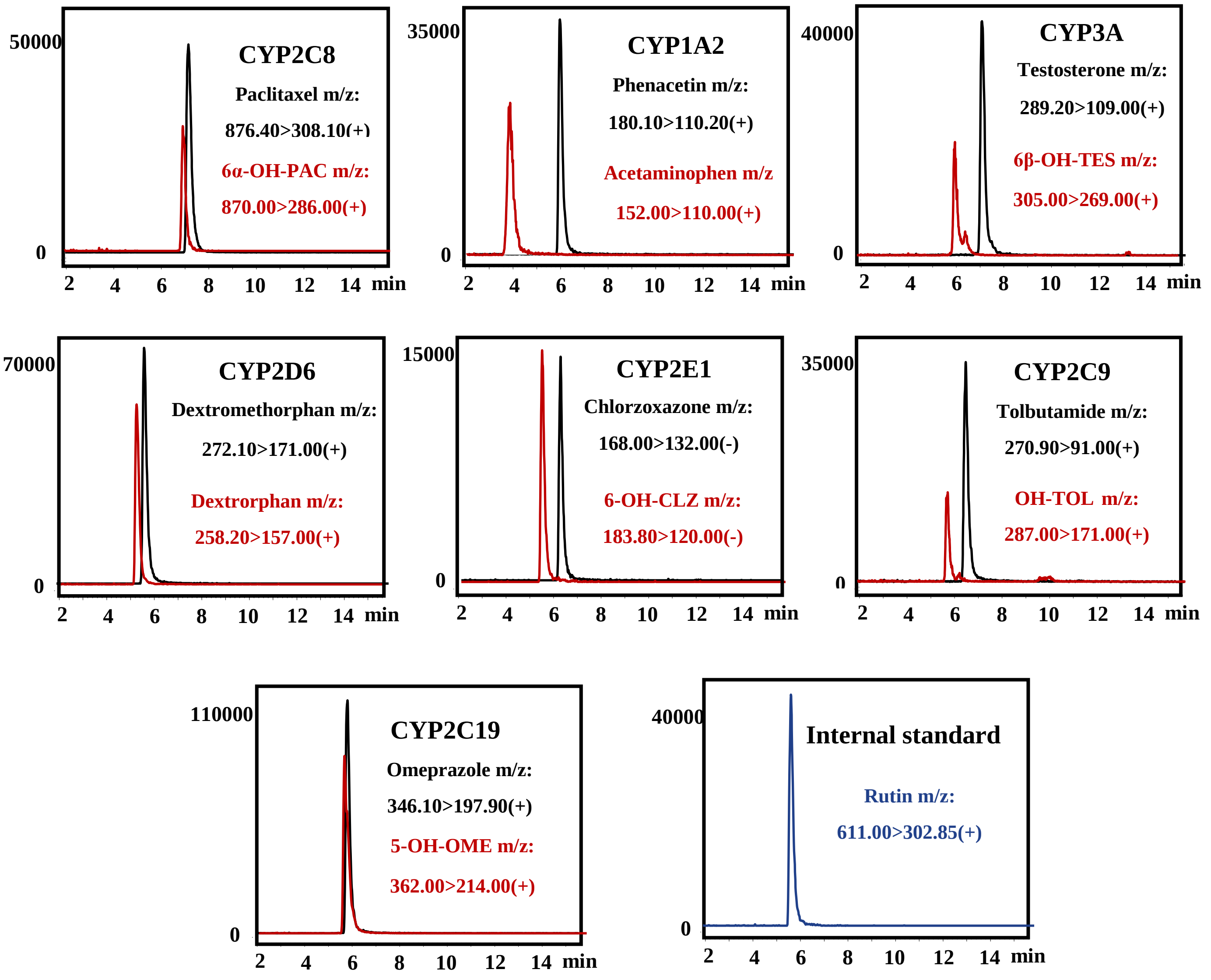

Supplementary FIGURE S3

Trend diagram of the generation of metabolites of seven enzyme substrates with time. (n=3)

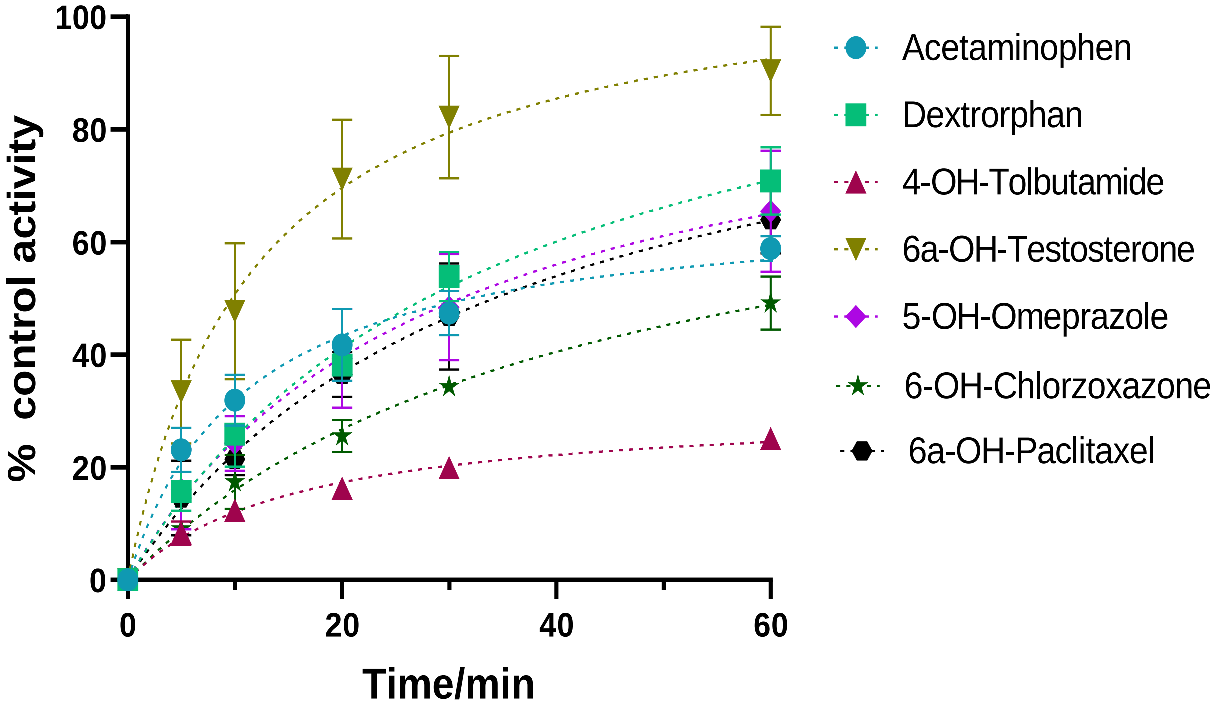

Supplement: Supplementary file 1 [file DataSheet1.pdf]
